# Clinical Efficacy and Future Prospects of Immunotherapy in Lung Cancer

**DOI:** 10.3390/life11101029

**Published:** 2021-09-30

**Authors:** Tomonari Kinoshita, Hideki Terai, Tomonori Yaguchi

**Affiliations:** 1 Division of General Thoracic Surgery, Department of Surgery, School of Medicine, Keio University, Tokyo 160-8582, Japan; 2 Division of Pulmonary Medicine, Department of Medicine, School of Medicine, Keio University, Tokyo 160-8582, Japan; hidekiterai@keio.jp; 3 Center for Cancer Immunotherapy and Immunobiology, Department of Immunology and Genomic Medicine, Graduate School of Medicine, Kyoto University, Kyoto 606-8501, Japan; yaguchi.tomonori.4m@kyoto-u.ac.jp

**Keywords:** lung cancer, immunotherapy, immune checkpoint inhibitor

## Abstract

The three major conventional treatments: surgery, chemotherapy, and radiation therapy, have been commonly performed for lung cancer. However, lung cancer is still the leading cause of cancer-related mortality. Immunotherapy has recently emerged as a very effective new treatment modality, and there is now growing enthusiasm for cancer immunotherapy worldwide. However, the results of clinical studies using immunotherapy are not always favorable. Understanding the steps involved in the recognition and eradication of cancer cells by the immune system seems essential to understanding why past immunotherapies have failed and how current therapies can be optimally utilized. In addition, the combination of immunotherapies, such as cancer vaccines and immune checkpoint inhibitors, as well as the combination of these therapies with three conventional therapies, may pave the way for personalized immunotherapy. In this review, we summarize the results of immunotherapies used in phase III clinical trials, including immune checkpoint inhibitors, and discuss the future prospects of immunotherapies in lung cancer treatment.

## 1. Introduction

Lung cancer is one of the most deadly of solid cancers [1]. Advanced lung cancer is a prevalent disease with high mortality and low response to conventional cytotoxic therapies. Lung cancer is classified into different histological types, such as adenocarcinoma, squamous cell carcinoma, large cell carcinoma (commonly referred to as non-small cell lung cancer (NSCLC)), and small cell lung cancer (SCLC), and the treatment strategy varies depending on the histological type, as well as the degree of progression. The recommended medical management for most patients with advanced stage NSCLC, mainly chemotherapy, improved progression-free survival (PFS) of 4–6 months and overall survival (OS) of about 12–18 months [2,3]. PFS and OS have been marginally improved by newer agents such as angiogenesis inhibitors, such as bevacizumab [4] and targeted chemotherapy based on oncogenic mutations [5]. Recently, immunotherapeutic agents targeting immune checkpoint pathways have shown great promise in clinical trials and are rapidly being incorporated into the standard of care for advanced stage NSCLC. We will review the results of clinical phase III trials of immunotherapy in lung cancer reported so far and discuss the future prospects of immunotherapy.

## 2. Initial Clinical Trials of Immunotherapies in Lung Cancer

Currently, there is no doubt that immune checkpoint inhibitors (ICI) are the mainstay of current immunotherapy in lung cancer. In particular, the results of the ICI clinical trials in 2015 (nivolumab versus. Docetaxel, CheckMate 017 and 057) were sensational [6,7]. Before the emergence of ICI, various immunotherapies, such as cancer vaccine therapy and cytokine therapy were tested in clinical trials. In this section, we will describe phase III clinical immunotherapy trials for lung cancer conducted before 2015, in order of the year they were reported.

### 2.1. Cancer Vaccine Therapies (including Cancer Antigen-Related Vaccine Therapies)

Antigen-specific vaccines are intended to induce specific anti-tumor immunity against specific tumor-associated antigens (TAAs). Conversely, tumor cell vaccines, consisting of autologous or allogeneic tumor cells, often expose the immune system to a variety of unknown tumor antigens [8]. Several lung cancer vaccine therapies have been performed in clinical trials; however, these results are not always favorable [9].

A phase III trial of MVP (mitomycin, vindesine, and cisplatin) with or without monthly mycobacterium vaccine administration (SRL172) for advanced NSCLC was performed and showed no survival benefit in 2004 [10]. A phase III trial of Bec2/BCG vaccine as maintenance therapy for localized SCLC treated with chemoradiation (vaccine arm versus observational arm) also failed to show prolonged survival in 2005 [11,12].

Ten years later, the results of clinical trials using other cancer vaccines were also published. In 2014, a placebo-controlled phase III trial of a vaccine targeting a major related ganglioside (Racotumomab-Alun) as maintenance therapy for advanced NSCLC previously treated with platinum was conducted in Cuba and showed advantage in survival rate of both PFS and OS, although it was suggested that the low rate of subsequent therapy may have influenced the results [13]. In addition, a placebo-controlled phase III study of a mucin 1 (MUC1) antigen-specific cancer vaccine (L-BLP25) after chemoradiation in unresectable stage III NSCLC did not show survival superiority [14].

Patients with NSCLC which express TAA melanoma-associated antigen-A3 (MAGE-A3) are often associated with poor prognosis. The MAGE-A3 vaccine is a combination of recombinant MAGE-A3 protein and the immunostimulant AS02B. It was evaluated in a large, double-blind, randomized phase III trial (MAGRIT) in 2015. This trial had negative results. There was no improvement in disease-free survival (DFS) in the vaccine group compared to placebo, either in the overall population or in patients who did not receive chemotherapy [15]. The clinical application of belagenpumatucel-L, produced by cryopreservation of four NSCLC cell lines with suppressed transforming growth factor-β (TGF-β) production after radiotherapy, also did not show superiority in both PFS and OS [16].

### 2.2. Activation of Immune System through Toll-like Receptor

Agonist antibody to costimulatory molecule or costimulatory receptor was clinically used in clinical trials as a promising immunotherapy. A phase III trial of cisplatin plus carboplatin plus paclitaxel in 2011 [17] and gemcitabine in 2012 [18] or plus a Toll-like receptor 9 agonist (PF3512676) for advanced NSCLC did not show a survival advantage in the initial interim analysis and was discontinued due to a high number of adverse events (injection-site reaction, neutropenic, and septic events).

### 2.3. Other Immune-Related Therapies

A phase III trial of cisplatin plus gemcitabine with or without low-dose IL-2 for advanced NSCLC did not show advantage in survival rate on 1-year survival rate in 2011 [19]. Other reports are also searching results from phase II trials, however, cytokine therapy has not been established yet [20,21]. A phase III trial investigating the efficacy of platinum combination therapy and activated killer T-cells/dendritic cells (DCs) as postoperative adjuvant chemotherapy has shown prolonged survival in an interim analysis in 2015, but there are problems such as the inclusion of stage IV patients, which is different from conventional adjuvant chemotherapy [22]. Talactoferrin alpha, a recombinant human lactoferrin, is an orally available dendritic cell-mediated immunotherapeutic agent. It interacts with DCs in the intestinal wall and is thought to stimulate migration and maturation of DCs as tumor antigen-presenting DCs [23,24,25]. Antitumor effects were observed in various preclinical models [26,27]. The FORTIS-M study was performed which is an international randomized trial compared talactoferrin alfa with placebo in patients with advanced NSCLC who had failed two or more prior therapies. Unfortunately, there was no statistical difference between talactoferrin-administered between placebo-treated group [28].

A meta-analysis of cancer vaccine therapy in NSCLC reported that both PFS and OS were prolonged, however, the stage of the patients was different. Therefore, the results of the analysis need to be understood with caution [29]. Considering the results of immune-related clinical trials, including Toll-like receptor agonist treatment trials, the clinical efficacy was not as good as expected, and immunotherapy for lung cancer has not received much attention.

## 3. Rise of Immune Checkpoint Inhibitors in Lung Cancer Treatment

Immune checkpoints are proteins on the surface of T-cells and other immune cells that function as negative regulators of immune activation by a variety of antigens, including tumor antigens. ICIs are a class of immunotherapeutic agents that harness the intrinsic immune response to tumor antigens by removing the brakes on T-cell activation by antigen-presenting cells (APCs). The first checkpoint discovered was cytotoxic T-lymphocyte antigen-4 (CTLA-4), and ipilimumab, an ICI developed to target CTLA-4, has shown prolonged survival in melanoma [30]. Such ICI-mediated activation of anti-tumor activity has shown great promise in other tumor types as well. For example, treatment with ICIs targeting CTLA-4 or another immune checkpoint, programmed cell death 1 (PD-1), has been shown to be effective in advanced melanoma [31,32], colon cancer with mismatch-repair deficiency [33], Hodgkin lymphoma [34], and renal cell carcinoma [35].

Among the various checkpoint pathways, the PD-1 pathway, consisting of the receptor (PD-1) and its reciprocal ligands (programmed death-ligand 1/2 (PD-L1 and PD-L2, respectively)), CTLA-4 pathway, have been most intensely studied in NSCLC in recent years. Monoclonal antibodies targeting PD-1 (e.g., nivolumab, pembrolizumab, sintilimab, cemiplimab), PD-L1 (e.g., atezolizumab, durvalumab), or CTLA-4 (ipilimumab), have been investigated in clinical trials for lung cancer and have demonstrated significant improvements in survival.

### 3.1. Nivolumab

Clinically, nivolumab showed an OS advantage over docetaxel, the conventional standard of chemotherapy in 2015. Of note, there is the tail plateau effect seen in nivolumab-treated patients, in which the OS and PFS curves almost cease to decline after a certain point, indicating a long-term progression-free survival effect that was thought to be impossible to achieve with conventional therapy (CheckMate 017, 057) [6,7]. The phase III Checkmate 816 trial is currently underway and is attempting to compare nivolumab plus two platinums with chemotherapy as neoadjuvant therapy for resectable NSCLC (stage IB-IIIA).

### 3.2. Pembrolizumab

Pembrolizumab is the most widely used ICI in lung cancer currently. KEYNOTE-010 trial showed the efficacy of pembrolizumab for patients with previously treated, PD-L1-positive, advanced NSCLC [36]. Furthermore, the efficacy of single-agent pembrolizumab as a first-line treatment for NSCLC with high PD-L1 expression was demonstrated (KEYNOTE-024) [37,38]. OS improvement after pembrolizumab alone treatment was investigated in patients with PD-L1 of 1% or higher to reconfirm its efficacy (KEYNOTE-042) [39]. Another comparative phase III study of platinum-based pemetrexed plus pembrolizumab in chemotherapy-naive advanced non-squamous NSCLC without EGFR or ALK mutations showed prolonged OS regardless of PD-L1 expression status (KEYNOTE-189) [40]. For squamous cell carcinoma, a comparative phase III study of platinum plus paclitaxel plus pembrolizumab showed an add-on effect both on OS and PFS (KEYNOTE-407) [41].

### 3.3. Sintilimab

A randomized, double-blind, phase III trial of sintilimab (fully human anti-PD-1 antibody) with pemetrexed and platinum was conducted in China. In Chinese patients with previously untreated locally advanced or metastatic non-squamous NSCLC, the addition of sintilimab to pemetrexed and platinum-based chemotherapy significantly prolonged PFS with a manageable safety profile compared to chemotherapy alone (ORIENT-11) [42]. In the second report of this study, it was shown that high expression of major histocompatibility complex (MHC) class II, as well as high immune cell infiltration could be predictive biomarkers in this sintilimab trial [43].

### 3.4. Cemiplimab

In the first-line treatment of advanced NSCLC with PD-L1 expression rates of 50% or higher, cemiplimab monotherapy significantly improves OS and PFS compared with chemotherapy. As well as sintilimab, cemiplimab may provide a new treatment option for patients with high PD-L1-expressing NSCLC.

### 3.5. Atezolizumab

The IMPower110 trial showed that atezolizumab alone significantly prolonged OS compared to platinum-based chemotherapy in patients with NSCLC of any histology with high expression of PD-L1 [44]. In 2019, IMPower130 study, which was designed to evaluate the efficacy and safety of the combination of atezolizumab and chemotherapy (carboplatin and nab-paclitaxel) versus chemotherapy alone as first-line therapy for non-squamous NSCLC, showed survival improvement of OS and PFS [45]. On the other hand, atezolizumab to platinum (carboplatin or cisplatin) plus pemetrexed in chemotherapy-naive patients with advanced non-squamous or NSCLC showed an add-on effect not on OS but on PFS in the phase III study in 2020 (IMPower132) [46]. In the first-line treatment of stage IV squamous NSCLC, the addition of atezolizumab to platinum-based chemotherapy significantly improved PFS, but not OS (IMPower131) [47].

The combination of atezolizumab with paclitaxel plus bevacizumab in chemotherapy-naive patients with metastatic non-squamous NSCLC patients showed its additional affect in IMPower150 study [48,49]. However, in the final analyses of IMPower150, the additional effect of atezolizumab resulted in a numeric, but not statistically significant, improvement in OS. This may be correlated with PD-L1 expression on tumor cells [50].

Atezolizumab was shown to be effective and safe as neoadjuvant immunotherapy for stage IB-IIIB resectable NSCLC in the phase II trial (LCMC3) [51]. In addition, many clinical trials are currently underway for preoperative therapy using ICIs (NCT02818920, NCT03425643, NCT02259621, NCT03081689, NCT02998528, NCT03158129, NCT03456063, NCT02927301). Moreover, for SCLC, the IMpower133 study, which aimed to evaluate the add-on effect of atezolizumab to carboplatin plus etoposide in chemotherapy-naïve SCLC patients, showed a significant improvement in OS (median OS was 12.3 with atezolizumab and 10.3 months with placebo). This was very sensational news in the treatment of SCLC, which has remained unchanged from the current standard of care for over 20 years. It is clinically meaningful and will be a new standard treatment option [52].

### 3.6. Durvalumab

A phase III study comparing durvalumab and placebo in patients with non-resectable, locally advanced NSCLC who were progression-free after concurrent chemoradiation showed significant improvement in OS and PFS [53,54].

### 3.7. Ipilimumab

Based on the results of CheckMate 227, nivolumab plus ipilimumab is recommended as first-line therapy for NSCLC with metastatic disease, regardless of PD-L1 expression, although the amount of tumor mutation burden (TMB) may be related [55] (discussed below). Additionally, it was investigated whether the addition of two cycles of chemotherapy to this combination would further enhance the clinical benefit. The combination of nivolumab and ipilimumab plus two cycles of chemotherapy resulted in a significant improvement in overall survival and a favorable risk-benefit profile compared with chemotherapy alone (Checkmate 9LA) [56]. In addition, as mentioned above, the efficacy of single-agent pembrolizumab as first-line therapy for NSCLC with high PD-L1 expression has been demonstrated [37,38], however, the KEYNOTE-598 trial, which aimed to show an add-on effect of ipilimumab to pembrolizumab, failed to demonstrate such an effect and was terminated [57]. A phase III study of the add-on effect of ipilimumab to platinum plus etoposide in SCLC also failed to show OS benefit [58].

## 4. Aiming for Individualized Immune Checkpoint Inhibitor

### 4.1. Detection of PD-L1 Expression

To date, several biomarkers for ICI have been reported. PD-L1 immunohistochemistry (IHC) was the first FDA-approved companion diagnostic test for ICI. IHC assays are performed using standard methods used in other IHC companion diagnostic tests, such as hormone receptor expression in breast cancer [59] and is read by a pathologist to estimate the percentage of tumor cells with membranous expression intensity and the percentage of immune cells with similar expression. Currently, there are four PD-L1 assays approved by the FDA for lung cancer. Several studies have compared the sensitivity and reproducibility of these assays to detect PD-L1 expression in both tumor cells and immune cells [60]. Only two of these studies, one sponsored by the National Comprehensive Cancer Network and the other by the Blueprint Project, have been prospectively statistically corroborated. The main conclusions of these studies were unanimous: SP142 is less sensitive than other FDA-approved assays and popular laboratory-developed assays, and pathologists can read PD-L1 expression in tumor cells but cannot read PD-L1 expression in immune cells in agreement [61,62]. The 22C3 assay has been approved by the FDA as a companion diagnostic to pembrolizumab, while other assays have been approved as complementary [63]. There are limitations to the predictive properties of these assays. This is because a benefit may be seen in patients whose tumors do not express PD-L1, and patients whose tumors express PD-L1 may not benefit from PD-(L)1 axis ICIs. The reason for this somewhat confusing result is the non-specificity of the IHC test. International association for the study of lung cancer (IASLC) pathology committee group provided updates on the indications of ICIs for lung cancer in 2019 and discussed important considerations on pre-analytical, analytical, and post-analytical aspects of PD-L1 IHC testing [64]. They stated that in addition to methods for assessing PD-L1 expression, the need for training of pathologists, methods for creating standardized reports and rules for interpretation should also be established in the future. However, for the time being, PD-L1 can be and should be used as a biomarker to broadly indicate response to ICI and to select patients who can receive single-agent pembrolizumab as first-line therapy for advanced non-squamous NSCLC.

### 4.2. Tumor Mutation Burden

In recent years, TMB has been paid attention to as another indicator of response to ICIs. The premise of this test is that as the number of non-synonymous mutations increases, a unique tumor neoantigen is generated that is recognized by the immune system and promotes tumor recognition and killing by adaptive immune cells when reactivated by ICI [65,66]. This relationship has been demonstrated in retrospective analyses of clinical trials, such as the subset of patients treated in the POPLAR and OAK trials [67], as well as in retrospective non-clinical studies [68,69]. Currently, these data indicate clinical benefit with respect to overall response rate or PFS, but not OS. The association between mutational load and susceptibility to ICI is also evident in so-called highly mutated tumors in patients with adverse alterations in DNA repair genes, such as MLH1, MSH2, MSH6, and PMS2, characterized by increased CD8^+^ T-cell infiltration [70,71], as well as in BRCA2 [72], POLD1, and POLE [73].

In lung cancer, Rizvi and co-authors demonstrated TMB is a potential biomarker for immunotherapy [68]. However, actually, TMB has many limitations that make it still inadequate for use in routine clinical practice. For instance, long sequencing panels are required to evaluate TMB, which requires large amounts of tumor tissue. Another major obstacle to introducing TMB as a biomarker into routine clinical practice is the need to standardize the available tests to achieve a more homogeneous approach to measuring TMB levels and to ensure comparability between tests. In addition, despite the widespread use of terms such as “low TMB” and “high TMB,” there is no clearly established threshold for defining high or low levels [74].

Because many patients with advanced lung cancer are unable to obtain sufficient tumor tissue for molecular analysis, there is interest in identifying less invasive methods to guide immunotherapy, such as the use of circulating tumor DNA to detect TMB, known as blood TMB. Circulating tumor DNA-mediated measurement of TMB in peripheral blood appears to be a promising strategy for obtaining an effective and independent biomarker for estimating response to immunotherapy. However, many questions remain unsolved, such as appropriate panel size and which variants to include [75].

### 4.3. Tumor-Infiltrating T Cells

Measurement of T-cell and other immune cell populations in tumor tissue is being actively developed as a predictor and endpoint for pharmacodynamic biomarkers and post-operative survival. In a variety of tumor types, the presence of significant baseline T-cell infiltration and interferon gamma (IFNγ)-related mRNA signatures (indicative of increased adaptive anti-tumor responses) has consistently been associated with increased sensitivity to ICIs and improved prognosis [76,77,78,79,80]. In patients with advanced NSCLC, the detection of increased CD8^+^ tumor infiltrating lymphocytes (TILs) or CD8A mRNA transcription by IHC is associated with significantly prolonged PFS after treatment with PD-(L)1 inhibitor; this association was enhanced by combining PD-L1 with protein and mRNA levels, suggesting that the integration of these biomarkers may increase their predictive value [81]. In another study, which used multiplexed quantitative immunofluorescence to measure TILs in formalin-fixed, paraffin-embedded tumor specimens, the relationship between baseline CD3^+^ levels and clinical outcome was examined, and there was a significant association between sustained efficacy and OS [69]. In addition, this study identified a subset of high CD3^+^ T-cell-infiltrating tumors with limited sensitivity to ICI, characterized by low T-cell proliferation (Ki-67 in T-cells) and cytolysis markers (granzyme B in T-cells). On the other hand, ICI treated patients whose tumor contained high CD3^+^ T-cell with high Ki-67 and granzyme B have favorable prognosis, indicating that not all inflamed (hot) tumors are the same. Recently, similar findings were reported in patients with advanced solid tumors using an 18-gene inflammatory mRNA signature. The higher the inflammation score, the greater the sensitivity to pembrolizumab, but some tumors with higher scores were noticeably more resistant to treatment [82]. There are some points to keep in mind when examining the relationship between TIL assessment and prognosis in lung cancer. We have reported that the prognostic impact of TIL may vary depending on the histological type of lung cancer and the effect of smoking [83,84]. In NSCLCs except for adenocarcinoma, such as squamous cell carcinoma, most were affected by smoking, and high accumulation of CD8^+^ TIL was a factor for good prognosis in these tissues. However, in adenocarcinoma where the effect of smoking was not clear, CD8^+^ TIL did not correlate with prognosis, and FOXP3 positive T-cells correlated with prognosis. In particular, in adenocarcinomas of non-smokers, the high accumulation of CD8^+^ TIL was a factor in poor prognosis. These CD8^+^ T-cells were mainly CD8^+^ FOXP3^+^ T-cells and CD8^+^ GATA3^+^ T-cells, and the expression of CCR4 and CCL17 was also high in the tumor micro-environment. These results suggest that a negative immune environment is more likely to form in adenocarcinoma tissues of nonsmokers, which may be one of the reasons why ICI is less effective. Such analysis may also help to refine current strategies for individualized immunotherapy, including ICIs for NSCLC.

Although a variety of other biomarkers have been reported, the precise biomarker for ICI has not been identified at this time. Standardization of the best assays and platforms to find reliable predictive biomarkers for immunotherapy is ongoing, and prospective studies are needed to demonstrate its value.

## 5. Why Have Previous Immunotherapies Failed?

Nivolumab showed an OS and PFS advantage over docetaxel in 2015 [6,7]. This report was very sensational since the tail plateau effect was seen in nivolumab-treated patients. OS and PFS curves almost stop falling after a certain point, suggesting a long-term PFS effect which seemed to be impossible to achieve with conventional therapy, such as chemotherapy. However, in fact, about half of the patients got worse by 3 months after the beginning of treatment. This result suggests that the clinical efficacy of ICI in lung cancer can be divided into three types: long-term survivors whose survival curves form a tail plateau, primary resistant patients who show little clinical efficacy, and acquired resistant patients who become resistant after about one year of treatment.

In general, immunotherapy resistance is classified as primary resistance or acquired resistance [85]. Primary resistance, also known as intrinsic resistance, represents a clinical situation in which a malignant tumor does not respond to immunotherapy. Acquired or extrinsic resistance represents a clinical situation in which a tumor can initially respond effectively to immunotherapy but relapse or progress after a period of treatment. Because of the short duration of clinical use of ICIs and the limited number of patient samples to analyze the mechanisms of resistance, the molecular mechanisms that lead to acquired resistance to these agents remain unclear [86,87,88]. Loss-of-function mutations and homozygous deletions of b2-microglobulin (B2M) leading to defects in human leukocyte antigen 1 (HLA I) antigen presentation have been reported in tumors that are resistant to ICI and other immunotherapies, such as lung cancer, melanoma, and colorectal cancer [88,89,90,91,92]. Decreased HLA I antigen presentation without genetic loss is also possible, suggesting that mechanisms other than genomic alterations may lead to resistance [88]. Loss of HLA I antigen presentation in tumors can occur in a number of other ways. Antigen mutations and copy number loss in genomic regions encoding specific HLA alleles have been described as mechanisms of primary resistance to immunotherapy in several tumor types, including lung cancer [93,94]. Loss-of-function mutations in components of the interferon signaling pathway, such as JAK1/2, have been also identified as examples of primary resistance to ICI in melanoma [89], including upregulation of PD-L1 [95,96], loss of PTEN [97,98], loss-of-function mutations of STK11/LKB1 [99,100], activation of c-Myc [101], activation of the WNT/b-catenin pathway [102], and alterations in the chromatin regulator PBAF [103,104].

On the other hand, there are various immune and stromal cells and cytokines within tumor micro-environment, which can affect the response to immunotherapy. The changes of immunosuppressive cells, immunosuppressive cytokines, coinhibitory receptors, and costimulatory receptors in the tumor micro-environment can destroy the anti-tumor immune response, which are the important mechanisms mediating resistance to immunotherapy. Such acquired immune cell-dependent mechanisms have also been linked to ICI resistance, including insufficient T-cell activation [105], upregulation of additional immune checkpoints (such as TIM-3 and LAG-3) [88,106,107], and exclusion of T-cells from the tumor micro-environment due to lack of appropriate chemokines [108,109]. Other immune cell-related mechanisms including regulatory T-cells, myeloid-derived suppressor cells (MDSCs), tumor-associated macrophages (TAMs) are also known as extrinsic resistance to ICIs [85].

Other immunotherapies for lung cancer have also failed [110,111]. Reasons for failure include insufficient accumulation of mutated tumor antigens [112], limited cross-presentation due to suppressed migration of APCs from the tumor [113] or lack of necessary signals, such as CD40, which is delivered predominantly by CD4^+^ T-cells [114], and ineffective presentation of the lysed tumor cells used in the vaccine. The lysed tumor cells used in the vaccine were not presented effectively [115]. There may also be a limit to the number of CD8^+^ T-cells that will respond. This is one of the reasons adoptive immunotherapy using T-cells obtained from tumors was first used in lung cancer [116]. The failure of T-cells to migrate into tumors and the presence of local intratumor suppression are both well documented [117,118]. On the other hand, one way to balance inflammation and suppression is to alter the local cytokine environment. Cytokine therapy in lung cancer has failed, probably because the cytokine concentrations that can be achieved in the tumor after systemic administration are not sufficient to achieve this shift in balance. It is a more recent concept that there are limits to restimulation by APCs in tumors [119]. Finally, tumors can escape immunological selection by losing their antigens and antigen-presenting molecules, MHC class I [120].

In fact, it is important to obtain pre- and post-treatment tissue samples to determine what is happening in tumors that have become resistant to immunotherapy for lung cancer. There are a variety of approaches to studying the use of genetic testing to predict immunotherapy resistance. In NCT04300062, tumor tissues were collected at the time of progression during ICI therapy to ensure the study of molecular mechanisms involved in the progression of NSCLC and SCLC. By comparing the treatment outcomes of patients with specific molecular characteristics, NCT03512847 discovered a molecular profile that predicts resistance to immunotherapy and chemotherapy. Differences in molecular profiles before and after treatment may reveal mechanisms of resistance to treatment. A significantly higher likelihood of altered PD-L1 expression was observed in patients who received chemotherapy [121]. In the NCT04807114 study, tumor biopsies were taken from patients with advanced NSCLC prior to the start of treatment with immune checkpoint inhibitors to characterize the tumor micro-environment. They also profiled the immune composition of the peripheral blood. DARWIN II (NCT02314481) is an exploratory phase II trial examining the role of intratumor heterogeneity and the presence of neoantigens on the efficacy of anti-PD-L1 immunotherapy in patients with epidermal growth factor receptor (EGFR)- or HER2-mutated NSCLC who have relapsed after afatinib therapy. Investigators looked at whether intratumor heterogeneity was associated with PFS in patients who received atezolizumab or molecularly targeted therapy. NCT04405661 is an observational study that evaluated differences in genetic mutations and immune micro-environment in NSCLC patients with different responses to ICI therapy [122]. Thus, the mechanism of resistance to immunotherapy that occurs in lung cancer is still being investigated. In the future, clarification of the role and significance of resistance mechanisms will help to develop approaches to overcome or prevent the emergence of resistance mechanisms.

## 6. Promising Immunotherapies

Other immunotherapeutic approaches, including chimeric antigen receptor (CAR) and CD3-based bispecific agents, have been developed recently [123,124]. CAR is a synthetic molecule designed to direct T-cells to specific antigens expressed on the surface of tumor cells [125]. Unlike the physiological function of T-cells, CAR T-cells are able to recognize antigens independent of human leukocyte antigens (HLA) and have the ability to affect tumor cells with low HLA expression [126]. Tissue factor or coagulation factor III is overexpressed in many cancer types, including lung cancer [127]. In recent years, EGFR has been evaluated as a potential target for CAR T-cell therapy, and a correlation between infusion of CAR-T-EGFR cells and improved response rates has been found in the treatment of NSCLC [128]. Importantly, the CAR-T-EGFR protocol was safe and feasible for EGFR-positive advanced relapsed/refractory NSCLC, suggesting that CAR T-cell therapy may be a promising anticancer strategy for other solid tumors, especially those with high EGFR expression. Other commonly targeted antigens of NSCLC include mesothelin, MUC1, prostate stem cell antigen, carcinoembryonic antigen, PD-L1, CD80/CD86, inactive tyrosine-protein kinase transmembrane receptor, and human epidermal growth factor receptor 2 (HER2) [129].

An interesting aspect of adoptive immunotherapy for NSCLC is the use of glypican-3 (GPC3)-targeted T lymphocytes. Findings include, first, immunohistochemical assays showed that GPC3 was expressed in 66.3% of lung squamous cell carcinoma samples and 3.3% of lung adenocarcinoma samples, but not in normal lung tissue. Next, in two established lung squamous cell carcinoma xenograft models, CAR-GPC3 T-cells almost completely eliminated the proliferation of GPC3-positive cells, indicating that CAR-GPC3 T-cells are capable of detecting lung tumors and efficiently infiltrating cancerous tissues. GPC3 may be a promising target for the treatment of lung squamous cell carcinoma [130].

## 7. Translational Research

As described above, there are various limitations in the current immunotherapy for lung cancer. It is necessary to combine the current immunotherapy with basic research (translational research) so that it can be an effective treatment for more patients with advanced lung cancer. For example, the cGAS-STING pathway, one of the innate immune pathways, has been reported to be associated with ICI sensitivity, both at in vitro model and in pre-clinical models. Kitajima et al. reported that STING expression is low in lung cancer with both KRAS mutation and LKB1 loss (KL tumor), and that the low STING expression level is caused by methylation of the promoter region of the STING gene [131]. They showed demethylating agent can improve the efficacy of ICI in vivo KL tumor model. In addition, Sen et al. reported that STING expression is also repressed in SCLC and the use of DNA damaging agents may promote STING activation and increase the sensitivity to ICI in patients with SCLC [132]. Based on these results, several types of STING agonists are currently being tested in clinical trials (NCT02675439, NCT03956680, NCT03010176, NCT03249792, NCT04096638) [133].

## 8. Future Prospects of Lung Cancer Immunotherapy

An interesting, and potentially very attractive, new approach is to take a personalized approach to immunotherapy by harnessing the power of genetic sequencing. Although it is well known that cigarette smoking is associated with lung cancer carcinogenesis, there are many non-synonymous mutations in smoking-related lung cancers that have a molecular smoking signature [134], and can generate tumor-specific MHC class I restriction epitopes. Thus, every tumor is highly specific and has a unique antigen profile. This so-called tumor mutanome can be revealed by deep sequencing, and the immunogenicity of mutated peptides can be predicted in silico. These peptides can be used to track tumor-specific T-cell responses and can be incorporated into personalized vaccines [135,136]. Peptide neoantigen vaccines may not be sufficient to induce an effective tumor-specific immune response, and a combination therapy approach (e.g., ICI) may be required [9,137].

Successful immunotherapy of lung cancer requires a better understanding of immune escape and immunosuppression in lung cancer, and learning how to monitor immunity, edit immunity, and reactivate immunity in cancer. By learning to understand cancer immunosurveillance, immunoediting, and how to reactivate cancer immunity, we can embark on a future full of possibilities to use immunotherapy as a reliable lung cancer treatment. The logical combination of nivolumab and ipilimumab in melanoma and NSCLC looks promising, although there are considerable side effects [138]. Several studies have examined the efficacy of nivolumab in combination with various anticancer agents, including chemotherapy, molecular-target therapy, bevacizumab, and other immunotherapies [139]. T-cell immunoreceptor with Ig and ITIM domains (TIGIT) potently inhibits innate and adaptive immunity through a variety of mechanisms [140]. Blocking both TIGIT and PD-1/PD-L1 pathways enhances the expansion and function of tumor antigen-specific CD8^+^ T-cells [141,142]. In a phase I study of vibostolimab (an anti-TIGIT antibody) in patients with advanced NSCLC after ICI treatment, this treatment was well tolerated as monotherapy and showed moderate anti-tumor activity. A randomized phase II trial of tiragolumab, another anti-TIGIT antibody, in combination with atezolizumab showed a modest but significant improvement in PFS versus placebo plus atezolizumab (CITYSCAPE) [143]. Preliminary data from a clinical trial of LAG-3 antibody (liratrimab) in combination with nivolumab in melanoma patients suggested that it was more effective than nivolumab alone, suggesting that the anti-LAG-3 antibody may have clinical efficacy as a third ICI pathway following PD-1 and CTLA-4 [143]. There are also many clinical trials running to explore the efficacy of ICI as preoperative adjuvant therapies for lung cancer [51,144,145]. Epigenetic therapies can also be used to induce tumors to be more responsive to immunotherapy [146]. The long-term efficacy of ICIs is still unknown, and clinicians face the problems of how to identify patients for treatment such as biomarker issue and how to manage the toxicity that occurs and the cost of these therapies. We predict that future NSCLC treatments will include a combination of neoantigen vaccines and chemotherapy, as well as multiple ICIs.

## Data Availability

Not applicable.
